# Supplementary material for: Seabirds shaped the expansion of pre-Inca society in Peru
Source: PLoS One. 2026 Feb 11;21(2):e0341263. doi: 10.1371/journal.pone.0341263 (PMC12893552; doi:10.1371/journal.pone.0341263)
Supplement: S1 File — Text A. Avian zooarchaeology on the Peruvian southern coast. Text B. Maize isotope physiology. S1 Fig. Maize cobs from UC-008 Tomb 1 in the middle Chincha Valley, Peru. S2 Fig. Architectural friezes from major administrative sites in the Chincha and Pisco valleys depicting seabirds, fish, and possible sprouting maize. S3 Fig. Bivariate plots of wt% N and C/N atomic ratio vs. δ15N values of all archaeological maize from Chincha analyzed in this study, and bivariate plots of wt% N and C/N atomic ratio vs. δ15N values of archaeological maize from Chincha analyzed at a target weight of 1.0 mg and used for all statistical analyses. S4 Fig. Bivariate plots of δ34S vs. C:S ratios and δ34S vs. N:S ratios of all archaeological maize from Chincha analyzed in this study. S1 Table. Avifauna results from Jahuay, Cerro Azul and Lo Demás. S2 Table. Summary statistics for Chincha maize and comparative sample from Chile. S3 Table. Comparison of estimates of isotopic space size from standard ellipse area (SEA) and kernel utilization density (KUD). S4 Table. Pair-wise isotopic space overlaps from standard ellipse area (SEA) and kernel utilization density (KUD). (DOCX) [file pone.0341263.s001.docx]

**Supporting Information for**

**Seabirds shaped the expansion of pre-Inca society in Peru**

Jacob L. Bongers^1,2,3*^, Emily B. P. Milton^4,5,6**^, Jo Osborn^7^, Dorothée G. Drucker^8^, Joshua R. Robinson^9^, and Beth K. Scaffidi^10^

* Jacob L. Bongers (corresponding author)

Email: [jacob.bongers@sydney.edu.au](mailto:jacob.bongers@sydney.edu.au)

** Emily B. P. Milton (co-corresponding author)

Emily: [miltone2@msu.edu](mailto:miltone2@msu.edu)

Supporting Information

**Text A. Avian zooarchaeology on the Peruvian southern coast**

To assess the ubiquity of guano birds within zooarchaeological assemblages on the Peruvian south coast, we surveyed published faunal reports from LIP and LH contexts for the presence of seabirds. Published studies are limited, often listing avifauna as unidentified. Supplementary Table 1 summarizes avifauna results from three studies of two south coast sites: Cerro Azul, Cañete [1,2] and Lo Demás, Chincha [3]. Additionally, we present data from LIP and LH contexts at Jahuay (Topará Quebrada) [4], which are reported here for the first time. Jahuay and Cerro Azul contained both LIP and LH occupations, while Lo Demás has a confirmed LH occupation. While we determined the periods of certain individual contexts, it is difficult to differentiate pre-Inca from Inca period contexts in this region [3]. The original analysts were not certain of the date for every context. As such, the data summarized here represent both LIP and LH avifaunal exploitation. These assemblages also contained remains from fish, marine invertebrates, marine mammals (pinnipeds, cetaceans) and terrestrial mammals (camelids, *Cavia sp*., and domestic dogs). These taxa did not form part of the present analysis.

Many factors can contribute to differences in the taxonomic identification of zooarchaeological assemblages, especially fragmentation, access to and completeness of modern reference collections, and inter-observer error. Therefore, different analysts may identify fauna at different levels of taxonomic specificity (e.g., *Sula sp*. vs *Sula variegata*), complicating comparisons across different published assemblages. To address this, Supplementary Table 1 reports avifauna from these assemblages at the Family level, while also listing more specific identifications provided across the analyses. While this approach does render these results less taxonomically specific, we note also that Indigenous Andean classifications differed from Western taxonomy. It is possible that pre-Hispanic residents of Chincha did not recognize differences between some of these taxa, or that they divided into two or more categories birds which western scientists would class as a single species.

For each assemblage, we report the number of identified specimens (NISP) for each taxon, the number of taxa identified (NTAXA), and the relative abundance of each taxon. We assigned each taxon a rank based on its relative abundance. Cormorants (Phalacrocoracidae) are consistently the highest-ranked taxon across all assemblages, comprising between 52.41-63.47% of the NISPs. Boobies and gannets (Sulidae) are the second-most abundant bird taxon, ranking either 2^nd^ or 3^rd^ for all individual assemblages and 2^nd^ across the collective assemblage. *Sulidae* made up between 7.31-19.88% of site-specific NISPs. The 3^rd^ most common taxon were Pelicans (Pelecanidae), comprising 14.21% of all identified specimens, and between 7.31-20.45% of site-specific NISPs. Gulls and terns (Laridae) were also identified relatively frequently across all four assemblages.

Differences in the relative abundance of various avian taxa across south coast sites may be due to a multitude of factors, including differences in site function, poor preservation of low-density skeletal elements, and heterogenous distribution of bird populations across the coast. We cannot discard the possibility that the high frequency of guano birds in archaeological assemblages is a factor of their overall abundance within south coast avian populations, rather than a reflection of coastal populations’ preferential taste for these birds. Regardless, the zooarchaeological data indicate consumption of guano birds and other seabirds among pre-Hispanic Andean societies on the coast.

**Text B. Maize isotope physiology**

C_4_ plants tend to be more enriched in ^13^C relative to C_3_ plants with maize averaging around -9 to -13‰. Unfertilized *δ*^15^N ranges for maize are variable (-0.3‰ to +5.7‰) and may reflect variations in soil composition, aridity, and other fractionation processes[5]. While *δ*^13^C values in maize are not significantly affected by guano application[6,7], fertilizers may alter modern maize cultivar *δ*^15^N values by as much as +40‰[5]. Unfertilized intra-plant variation in *δ*^15^N is low (<5‰) whereas manure-fertilized maize may vary significantly (up to 10‰) among plant tissues. Data to inform on cob-grain offsets in *δ*^15^N and *δ*^34^S are limited, though existing data suggest Δ^13^C_cob-grain_ and Δ^15^N_cob-grain_ are less than 1‰, regardless of soil amendments [8].


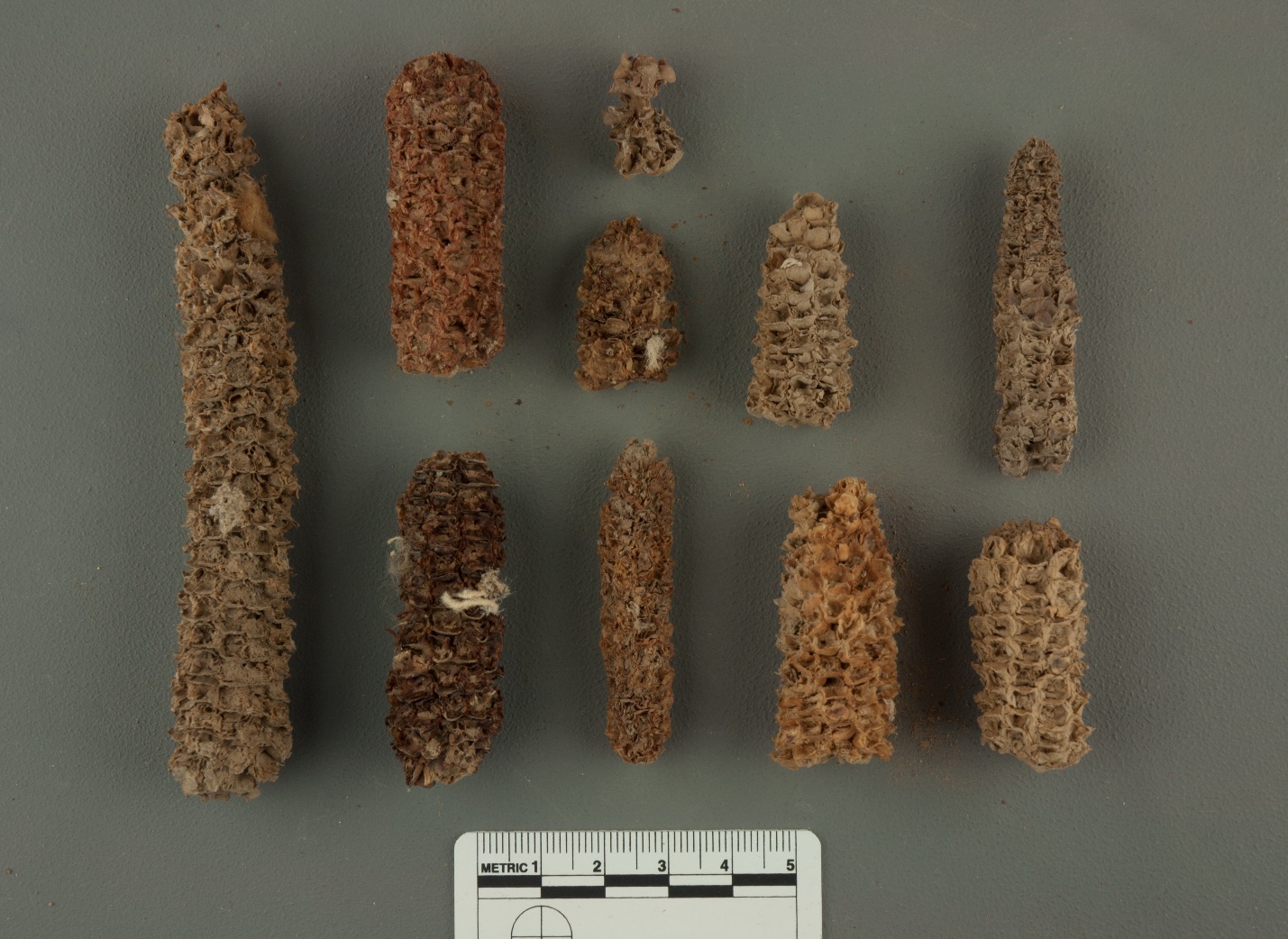


**S1 Fig.** **Maize cobs from UC-008 Tomb 1 in the middle Chincha Valley, Peru.** Photo by C. O’Shea.


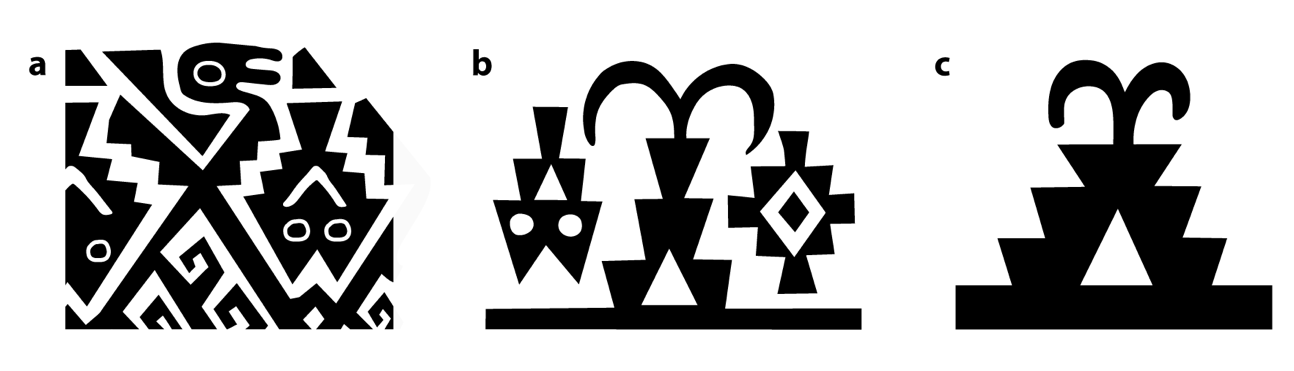


**S2 Fig.** **Architectural friezes from major administrative sites in the Chincha and Pisco valleys depicting seabirds, fish, and possible sprouting maize.** (a) La Centinela, Chincha Valley (as seen in Fig. 3*H*); (b) Litardo Bajo, Chincha Valley [9]; (c) Tambo Colorado, Pisco Valley [9].


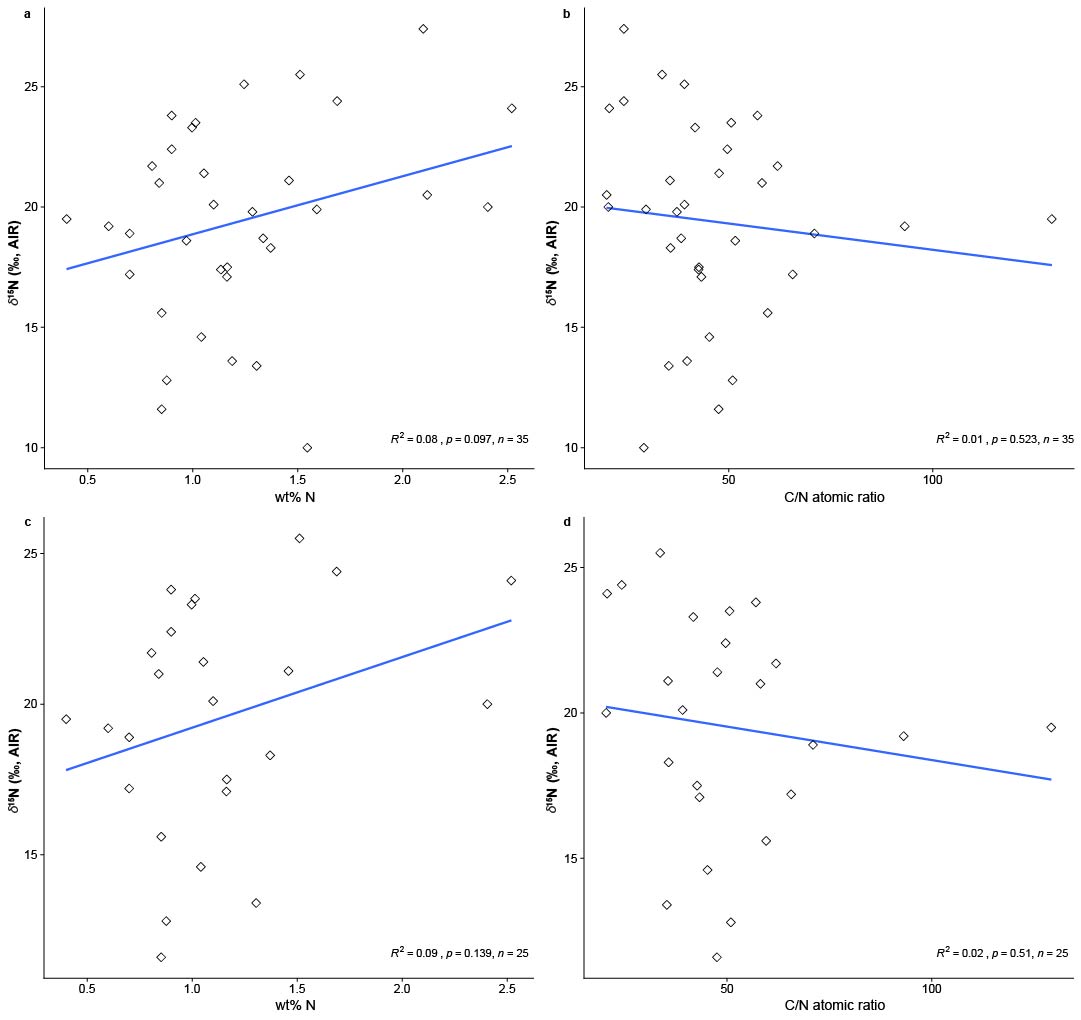


**S3 Fig.** **Bivariate plots of wt% N (a) and C/N atomic ratio (b) vs. δ^15^N values of all archaeological maize from Chincha analyzed in this study, and bivariate plots of wt% N (c) and C/N atomic ratio (d) vs. δ^15^N values of archaeological maize from Chincha analyzed at a target weight of 1.0 mg and used for all statistical analyses.** It has been suggested that a comparison of δ^15^N of plants with their C/N ratios may provide a check on whether very low nitrogen to high carbon concentrations produce abnormally high δ^15^N values [10]

**S4 Fig. Bivariate plots of (a) δ^34^S vs. C:S ratios and (b) δ^34^S vs. N:S ratios of all archaeological maize from Chincha analyzed in this study.**Some studies [11] have suggested that N:S ratios >20:1 in maize could indicate sulfur deficiencies in the soil.

|  |  |  | **S1 Table. Avifauna results from Jahuay, Cerro Azul and Lo Demás.** | | | | | | | | | | | | | | |
| --- | --- | --- | --- | --- | --- | --- | --- | --- | --- | --- | --- | --- | --- | --- | --- | --- | --- |
|  |  |  | **Jahuay** | | | **Cerro Azul** [2] | | | **Cerro Azul** [1] | | | **Lo Demás** [3] | | | **TOTAL** | | |
|  |  |  | NISP | Rank | Relative Abundance (%) | NISP | Rank | Relative Abundance (%) | NISP | Rank | Relative Abundance (%) | NISP | Rank | Relative Abundance (%) | NISP | Rank | Relative Abundance (%) |
| Family | Reported Identifications | Common Name |  |  |  |  |  |  |  |  |  |  |  |  |  |  |  |
| Anatidae | Anas sp. | Duck |  |  |  |  |  |  | 4 | 8 | 1.83% |  |  |  | **4** | **8** | **0.43%** |
| Diomedeidae | Diomedea sp. | Albatross |  |  |  |  |  |  | 1 | 10 | 0.46% |  |  |  | **1** | **11** | **0.11%** |
| Laridae | Laridae, Larus sp., Larus balcheri, Larus modestus, Leucophaeus pipixcan | Gull, tern | 2 | 5 | 0.74% | 17 | 4 | 10.24% | 13 | 4 | 5.94% | 52 | 2 | 19.48% | **84** | **4** | **9.04%** |
| Scolopacidae | Scolopacid | Sandpiper |  |  |  |  |  |  | 11 | 5 | 5.02% |  |  |  | **11** | **6** | **1.18%** |
| Columbidae | Zenaida sp., Zenaida melod, Zenaida auriculata | Dove |  |  |  |  |  |  | 6 | 7 | 2.74% | 15 | 5 | 5.62% | **21** | **5** | **2.26%** |
| Rallidae | Fulica ardesiaca, Gallinula chloropus, Pardirallus sanguinolentus | Rail |  |  |  |  |  |  | 10 | 5 | 4.57% |  |  |  | **10** | **7** | **1.08%** |
| Pelecanidae | Pelecanus sp., Pelecanus thagus | Pelican | 55 | 2 | 20.45% | 29 | 3 | 17.47% | 16 | 2 | 7.31% | 32 | 4 | 11.99% | **132** | **3** | **14.21%** |
| Pelecanoididae | Pelecanoides garnotii | Peruvian diving petrel | 3 | 4 | 1.12% |  |  |  |  |  |  |  |  |  | **3** | **9** | **0.32%** |
| Procellaridae | Puffinus sp. | Shearwater |  |  |  |  |  |  | 1 | 10 | 0.46% |  |  |  | **1** | **11** | **0.11%** |
| Spheniscidae | Spheniscus humboldti | Humboldt penguin |  |  |  |  |  |  | 2 | 9 | 0.91% |  |  |  | **2** | **10** | **0.22%** |
| Phalacrocoracidae | Phalacrocoracidae, Leucocarbo Bougainvillii, Phalacrocorax sp., Phalacrocorax gaimardi | Cormorant | 161 | 1 | 59.85% | 87 | 1 | 52.41% | 139 | 1 | 63.47% | 140 | 1 | 52.43% | **527** | **1** | **56.73%** |
| Sulidae | Sulidae, Sula sp., Sula variegata | Gannet, Booby | 47 | 3 | 17.47% | 33 | 2 | 19.88% | 16 | 2 | 7.31% | 37 | 3 | 13.86% | **133** | **2** | **14.32%** |
|  |  | **TOTAL** | **268** | | | **166** | | | **219** | | | **276** | | | **929** | | |
|  |  | NTAXA |  | 5 |  |  | 4 |  |  | 11 |  |  | 5 |  |  | 12 |  |

**S2 Table. Summary statistics for Chincha maize and comparative sample from Chile** [12]**.** LIP = Late Intermediate Period; ND = no date/maize from undated context.

|  | Chincha | | | Chile | | |
| --- | --- | --- | --- | --- | --- | --- |
|  | *n* | *δ^13^C* | *δ^15^N* | *n* | *δ^13^C* | *δ^15^N* |
| Colonial | 2 | -9.9 ± 0.1 | 18.7 ± 2.1 | 0 | --- | --- |
| Inca | 2 | -10.1 ± 0.0 | 19.4 ± 0.2 | 19 | -10.7 ± 0.7 | 22.9 ± 4.2 |
| LIP | 4 | -9.9 ± 0.4 | 21.0 ± 3.3 | 48 | -10.2 ± 0.7 | 20.6 ± 6.0 |
| pre-LIP | 0 | --- | --- | 34 | -10.6 ± 0.9 | 7.3 ± 5.2 |
| ND | 18 | -9.9 ± 0.8 | 19.3 ± 4.4 | 0 | --- | --- |
| Total | 25 | -9.9 ± 0.7 | 19.5 ± 3.8 | 101 | -10.4 ± 0.8 | 16.6 ± 8.6 |

**S3 Table.** **Comparison of estimates of isotopic space size from standard ellipse area (SEA) and kernel utilization density (KUD).** Estimates of isotope space provided at 75% and 95% contour intervals.

|  | SEA | | KUD | |
| --- | --- | --- | --- | --- |
| **Fertilizer** | *75%* | *95%* | *75%* | *95%* |
| Ammonium sulfate | 17.89 | 38.66 | 25.52 | 47.18 |
| Camelid dung | 44.53 | 96.22 | 45.81 | 86.17 |
| Seabird guano | 137.60 | 297.36 | 165.09 | 317.19 |
| Unfertilized | 15.83 | 34.21 | 19.45 | 38.50 |
| Chincha | 33.75 | 72.93 | 31.70 | 65.13 |

**S4 Table. Pair-wise isotopic space overlaps from standard ellipse area (SEA) and kernel utilization density (KUD).** Overlaps provided at 75% and 95% contour intervals. Bolded values indicate where overlap is estimated.

|  |  | Ammonium sulfate | | Camelid dung | | Seabird guano | | Unfertilized | | Chincha | |
| --- | --- | --- | --- | --- | --- | --- | --- | --- | --- | --- | --- |
|  | **Fertilizer** | *75%* | *95%* | *75%* | *95%* | *75%* | *95%* | *75%* | *95%* | *75%* | *95%* |
| SEA | *Ammonium sulfate* | --- | --- | **0.61** | **0.81** | 0.00 | **0.12** | **0.69** | **0.71** | 0.00 | 0.00 |
|  | *Camelid dung* | **0.24** | **0.32** | --- | --- | **0.05** | **0.49** | **0.21** | **0.31** | **0.01** | **0.19** |
|  | *Seabird guano* | 0.00 | **0.02** | **0.02** | **0.16** | --- | --- | 0.00 | **0.01** | **0.23** | **0.25** |
|  | *Unfertilized* | **0.78** | **0.80** | **0.59** | **0.88** | 0.00 | **0.13** | --- | --- | 0.00 | **0.03** |
|  | *Chincha* | 0.00 | 0.00 | **0.01** | **0.25** | **0.92** | **1.00** | 0.00 | **0.01** | --- | --- |
| KUD | *Ammonium sulfate* | --- | --- | **0.55** | **0.71** | 0.00 | **0.21** | **0.69** | **0.70** | 0.00 | **0.05** |
|  | *Camelid dung* | **0.31** | **0.39** | --- | --- | **0.01** | **0.52** | **0.25** | **0.37** | **0.02** | **0.17** |
|  | *Seabird guano* | 0.00 | **0.03** | 0.00 | **0.14** | --- | --- | 0.00 | **0.04** | **0.16** | **0.21** |
|  | *Unfertilized* | **0.90** | **0.86** | **0.59** | **0.83** | 0.00 | **0.30** | --- | --- | 0.00 | **0.12** |
|  | *Chincha* | 0.00 | **0.04** | **0.03** | **0.22** | **0.85** | **1.00** | 0.00 | **0.07** | --- | --- |

**References**

1. Marcus J, Glew CP. The bird life of Cañete and the avifauna of Cerro Azul. In: Marcus J, editor. Coastal Ecosystems and Economic Strategies at Cerro Azul, Peru: The Study of a Late Intermediate Kingdom. Ann Arbor: The University of Michigan Museum of Anthropology; 2016. pp. 172–185.

2. Núñez Aparcana B, Avila Peltroche M, Castillo Sanchez N. Change and Continuity in the Diet of El Huarco–Cerro Azul, Peru, during the Inca Arrival (AD 1470–1532). In: Elkin D, Delaere C, editors. Underwater and Coastal Archaeology in Latin America. Gainesville: University of Florida Press; 2023. pp. 126–137.

3. Sandweiss DH. The Archaeology of Chincha Fishermen: Specialization and Status in Inka Peru. Pittsburgh: Carnegie Museum of Natural History; 1992.

4. Osborn J. Jahuay: maritime specialization in a littoral Topará community (200 BC–AD 150). Unpublished PhD dissertation, University of Michigan. 2022.

5. Szpak P, Longstaffe FJ, Millaire J-F, White CD. Stable isotope biogeochemistry of seabird guano fertilization: results from growth chamber studies with maize (Zea mays). PLoS One. 2012;7: e33741.

6. Metcalfe JZ, Mead JI. Do uncharred plants preserve original carbon and nitrogen isotope compositions? J Archaeol Method Theory. 2019;26: 844–872.

7. Szpak P, White CD, Longstaffe FJ, Millaire J-F, Vásquez Sánchez VF. Carbon and nitrogen isotopic survey of northern Peruvian plants: baselines for paleodietary and paleoecological studies. PLoS One. 2013;8: e53763.

8. Killian Galván V, Oliszewski N, Olivera D, Panarello H. Intraspecific variability in the δ13C and δ15N values of archaeological samples of Zea mays cobs (Northeastern Argentinean Puna). In: Kligmann D, Morales M, editors. Physical, Chemical and Biological Markers in Argentine Archaeology: Theory, Methods and Application. Oxford: BAR International Series 2678; 2014. pp. 39–51.

9. Morris C. Enclosures of Power: The Multiple Spaces of Inka Administrative Palaces. In: Evans ST, Pillsbury J, editors. Palaces of the Ancient World. Washington, D.C.: Dumbarton Oaks; 2004. pp. 299–323.

10. Szpak P, Chiou KL. A comparison of nitrogen isotope compositions of charred and desiccated botanical remains from northern Peru. Veg Hist Archaeobot. 2019; 1–12.

11. Sharma RK, Cox MS, Oglesby C, Dhillon JS. Revisiting the role of sulfur in crop production: A narrative review. J Agric Food Res. 2024;15: 101013.

12. Santana-Sagredo F, Schulting RJ, Méndez-Quiros P, Vidal-Elgueta A, Uribe M, Loyola R, et al. ‘White gold’ guano fertilizer drove agricultural intensification in the Atacama Desert from AD 1000. Nat Plants. 2021;7: 152–158.
