# Supplementary material for: Seabirds shaped the expansion of pre-Inca society in Peru
Source: PLoS One. 2026 Feb 11;21(2):e0341263. doi: 10.1371/journal.pone.0341263 (PMC12893552; doi:10.1371/journal.pone.0341263)
Supplement: Attachment — Submitted filename: Response to Reviewers.docx [file pone.0341263.s004.docx]

Responses to Feedback

*We appreciate the insightful feedback from the reviewers, which has improved the paper.*

**Reviewer #1:**

**The paper by Bongers et al presents new and original data about the possible fertilization of maize using seabird guano in the Chincha Valley (Peru) from the Late Formative until the Colonial Period. Even though this case seems to be a good example on the use of fertilizers in the Andean region, I am worried about certain methodological aspects that debilitate the study:**

**First of all, it is not clear to me why the authors analyzed surface-collected maize and not maize from their current or previous excavations in the cemeteries. The permanent exposure to sunlight, wind, dust, etc., could clearly affect the preservation of the archaeobotanical material. This is a very arid region and sun can affect negatively the maize conservation, cracking the cobs and allowing for contamination.**

*We understand the reviewer’s concern. All organic archaeological materials, regardless of depositional context, have the potential to undergo contamination after deposition. Typically, this is addressed via quality control screening measures. While quality control assessments for plant isotopic values remain an ongoing area of research, we have done our best to address diagenetic concerns throughout our methodology. We sampled from whole, visually intact maize cobs (no visible cracks), avoiding areas that appeared damaged or particularly dirty. We then applied the recommended cleaning protocol [1] for nitrate contaminants. We discuss these protocols in more detail in our main text and supplemental materials (lines 560-569; supplemental figure S3).*

**Usually when analyzing archaeological plants for stable isotope analysis is necessary to run separately carbon and nitrogen isotopes. This, because the amount of nitrogen in plants is considerable low and the nitrogen peaks in the IRMS will not be high enough, generating confusing data. In fact, after doing the carbon isotope run, it is possible to calculate the amount in mg that will be needed for the nitrogen run, making sure to obtain a decent nitrogen peak and good d15N values. By reading the materials and methods section of the paper, it seems that the authors did just one run (with duplicates and triplicates) for d13C and d15N. For me, this is complicated in terms of methodology, as I find it difficult to trust the data. Moreover, the amount of maize measured in the IRMS, approx 1 mg, is really low compared to what you would expect for an archaeobotanical dessicated plant (between 3-5 mg). In addition, maize is characterized by having a very low % of N, so it is compulsory to measure more sample (mg) to obtain good results. Would it be possible for the authors to re-analyze their samples by doing separate runs? This is of great relevance to give further support to their findings and interpretations.**

*We appreciate the reviewer’s concerns; however, we suggest that a re-analysis at higher masses is not presently substantiated. We applied published protocols from leading regional plant studies, such as Szpak and Chiou 2019 [2], who suggested a minimum threshold of 0.5 mg as sufficient for analysis. Given the differences in machinery and protocols among labs, we also relied on the expertise of the lab specialists handling our samples to ensure materials were prepared appropriately for analysis. For example, in our initial run, several samples weight at ~0.5 mg samples did not produce adequate peak areas (aligned with reviewer concerns). Following a consultation with the U.C. Merced lab director, these samples were re-run at higher masses (>1mg) with good results (see attached reports EA20231220_report_0.5mg and EA20240207_report_1.0mg, which detail this occurrence, as well as lab linearity corrections and peak information for our samples).*

*All samples analysed at ≥1 mg fell within accepted ranges for the machine sensitivity–with good peak areas and no apparent linearity effects–suggesting our masses were adequate for analysis. Prompted by the reviewer’s concerns, we consulted with additional lab personnel, including folk at the University of New Mexico Center for Stable Isotopes, regarding machine settings and appropriate masses for plant analysis. We were informed by both labs that only plants with very high C:N ratios, such as those typical of wood (200-1000), require independent measurements for nitrogen. Our sample C:N ratios do not meet this threshold. We were also informed that studies focused on nitrogen fixation, or like analyses requiring highly precise and accurate results, can request to analyse plants with lower C:N ratios in nitrogen-only mode; however, our research questions do not require this setting, as even a few per mille of inaccuracy would not change our interpretations. As sample mass appears to be an emerging area of concern for archaeologists, we will request our reviews are made open to provide full transparency to readers on our methodological decision making.*

**I do not understand why the authors analyze bird bone collagen. I think it is clear for the audience that these are marine birds and that they will definitely have high d15N values as they feed on fish (and fish from the Humboldt Current that will be even more enriched in 15N than other parts of the world). It is confusing and there is not a straight connection between the fertilizer findings with the zooarchaeological results. It doesn't make much sense to me. Actually, this data and its discussion seems a bit forced in the paper.**

*The bird bones provide a baseline for isotopic expectations, as the isotopic composition of marine and terrestrial ecosystems can vary significantly, even over short distances. As the reviewer notes, the highly productive Humboldt Current and associated marine ecosystem services are unique to the coast of South America; therefore, it is essential to establish isotopic expectations for the region and period, as we note on lines 114-115 and 207-210. As there are few d15N / d13C data, and almost no d34S data available for archaeological marine fauna in the Chincha area (or even Peru), we measured carbon, nitrogen, and sulfur isotopes from various bird bones as a means of establishing a baseline to estimate local isotopic variation within the guano system. We have added clarification that this was the reason for our study design on lines 370-372. We emphasize that our data reinforce the need for local baseline construction, as our sulfur values were ~5‰ lower than the generally accepted +20‰ cited for global reservoirs (see lines 376-378). As such, our data have helped establish a lower threshold for possible marine sulfur values.*

**On a different note, I find the structure of the paper somehow disorganized, especially in the discussion section. The authors jump from archaeological aspects to more isotope-methodological ones, to then go back to the archaeology discussion. I think the paper will be much better if the archaeology and isotope-methodology sections were separated and not mixing up along the discussion.**

*Thank you for this constructive recommendation. We have revised the Discussion section to improve clarity and structure. We now begin the section with a brief introduction outlining its focus on both methodological and empirical contributions. We have also separated the methodological and archaeological discussions into distinct subsections to enhance readability and ensure a more coherent flow of ideas.*

**I will suggest the authors to be a little more cautious on their interpretations about the use of seabird guano and its contribution to the development of the Chincha Kingdom. For sure this fertilizer had an enormous impact on the local populations. However, this study only considers 35 samples (surface collected). More samples are needed to make greater interpretations.**

*We are grateful to the reviewer for this important and thoughtful comment. While our results offer high-probability, multidisciplinary evidence for the use of seabird guano as a pre-Hispanic agricultural amendment, we agree that our sample size necessitates caution in drawing broader conclusions about the scale and sociopolitical impact of this practice within the Chincha Kingdom.*

*We have tempered our interpretations regarding the role of guano in the emergence of the Chincha polity. For example, in the Abstract on lines 47-48, we now, “suggest that seabird guano fertilization played an important role in the sociopolitical and economic expansion of the Chincha Kingdom.” In the Discussion, we qualify our interpretation by stating that guano, “likely constituted a critical source of economic and political power,” and, “may have played a key role” in local negotiations with the Inca Empire (lines 492, 512-513).*

**I honestly believe that by improving these aspects, the paper will make a stronger case study. I would like to highlight the importance of discussing the role of sulfur isotopes in crops, and what can they tell us about agricultural practices. Even though there is not much information about this in the literature, particularly in the Andes and dessicated crops, I congratulate the authors for exploring this new line of evidence.**

*It’s been a unique [challenging] experience to collect and situate new sulfur data within the regional context, so we sincerely appreciate the reviewer’s recognition of this component :)*

**Reviewer #2:**

**The manuscript by Bongers et al. "Seabirds Shaped the Expansion of Pre-Inca Society in Peru" asks a clear question: did seabird guano play a critical role in the agricultural and sociopolitical development of the Andes, particularly in the Chincha Valley?**

**The study is well written, has a compelling topic, and is methodologically robust. Its multidisciplinary approach, with topics as environmental history, archaeology and economic anthropology, is commendable, and the integration of stable isotope analysis into a broader historical and social framework is carefully executed.**

**However, the manuscript exaggerates the novelty of his contribution. It does not adequately situate its findings within the broader group of Andean archaeological and paleoecological studies. The manuscript should present a more balanced view, moderating claims to originality and engaging more deeply with the existing literature, especially given the well-established research on guano use, irrigation, and maize economies along coastal Peru. Although the authors claim that their study provides “the strongest evidence to date” for pre-Inca guano fertilization, they fail to acknowledge previous foundational work—notably that of Szpak (2014), Makarewicz & Tuross (2012), and others—that has already demonstrated the isotopic impact of guano on maize and human diets in Peru and Chile.**

*We appreciate the author’s perspective on the impact of our contributions, and have sought to moderate our claims, as we address to reviewer 1 above, and further, below. We also thank you for directing us to these references; Szpak’s graduate and subsequent work has been instrumental to development of isotopic fertilization studies in the region. We’ve added acknowledgement of these studies, which can be found on lines: 57-58, 100, 104*

**To strengthen the manuscript, authors should incorporate key references that provide critical context:**
**Chepstow-Lusty et al. (2009), which addresses agriculture as a central economic activity for Inca and Pre-Inca societies.**
**Snyder (2011), who discusses innovative irrigation techniques and their role in food security, including the use of guano.**
**Tykot and others. (2006), which links the introduction of corn to rapid population growth.**
**Rodrigues and Micael (2021), who highlight the role of guano birds in the expansion and prosperity of the Inca Empire and previous cultures.**

*We appreciate the reviewer’s concerns regarding the novelty of our argument and thank them for their provision of additional papers on this topic. We acknowledge (and cite) several studies that have conducted a multidisciplinary investigation of fertilization and have now sought to moderate our claims throughout the article. We feel our research is novel because we provide some of the first stable sulfur data for archaeological maize and guano birds in the Americas (previous studies have only contributed small (n=1) sample sizes with no quality control information). However, our argument about guano use does not rely solely on isotopic evidence, and we suggest our insights are unique for the south coast region; we consider geography (proximity to some of the most abundant guano deposits in the Andean region on the Chincha Islands) as well as archaeological (e.g., iconography, zooarchaeological remains, etc.) and historical (colonial-era documents) data reported in our paper. We agree with the reviewer that the paper would benefit from deeper engagement with the existing literature and are grateful for the thoughtful list of relevant sources on guano use and agricultural strategies. We have now cited all sources in the manuscript.*

**Although guano is emphasized, alternative nitrogen sources such as fish-based composts or legume rotation are not adequately considered. Addressing these alternatives is important to validate the distinct isotopic signal of guano.**

*We considered the possibility of other fertilizers but see this was not clear in our original text. As such, we’ve added a statement considering other fertilizers for our samples <20‰ (239-240). To date, guano is the only known amendment to produce >20‰ nitrogen values in agricultural crops (see lines 97-105 for more detail) (Szpak 2012a, 2012b, and 2014) [3–5], therefore, we still consider seabird guano as the only parsimonious explanation for many of our samples.*

**Although the discussion acknowledges the variability and limited understanding of sulfur uptake in plants, it still relies on δ³⁴S values to infer marine influence in paleodietary contexts. A stronger case would include experimental control data, such as fertilization trials under old analog conditions.**

*We agree on the need for additional analog trials; however, we suggest there is some urgency in publishing our current dataset, as is, and allowing experimental studies to follow. Stable sulfur is poised to become a new trend in the region, and as we outline repeatedly throughout the paper, should not be used uncritically (and merits more foundational work!).*

*Recent archaeological fertilization studies have increasingly noted a potential for "contamination” of terrestrial food systems (at the human and animal levels) by marine fertilizers; the implications of such contamination for paleodietary reconstructions of past agricultural communities are significant. Whether marine δ³⁴S is reliably routed into plants via fertilization is at the center of this discussion––as the common, two-isotope analyses of carbon and nitrogen have been implicated by the well-documented enrichment of 15N in marine-fertilized C4 plants.*

*If δ³⁴S values consistently increase to marine levels (≥+15‰) in terrestrial plants, we cannot reliably use δ³⁴S to discriminate between marine and terrestrial inputs in fertilizer-using periods. If, however, marine-fertilized plants do not consistently enrich in 34S––as existing experimental studies and our newly presented maize data suggest––then δ³⁴S may remain a reliable, and relatively simple/accessible method for better untangling human/animal foodwebs on the coast.*

*It would be a shame to throw the baby (δ³⁴S) out with the bathwater! As such, we’ve used our data to initiate this exact discussion, describing the complexity of the problem and emphasizing the criticality of more mechanistic studies on sulfur (lines 415-417; 430-432). Using our data and interdisciplinary interpretations, we hope to help direct people towards, dare we say, “fertile” areas of future research.*

**The manuscript does not sufficiently clarify the chronological precision or geographic specificity of the corn and guano samples. Without more precise dating, it is difficult to determine when guano fertilization became common in the Chincha Valley.**

*Thank you for requesting clarification regarding the chronology of our samples. As noted in the “Maize carbon and nitrogen isotopes” section (lines 225-226), unburned maize cobs are known to yield unreliable radiocarbon dates [6]. Since all of our maize samples are unburned, we chose not to date them directly. Instead, we dated associated materials—including tooth dentine, bone collagen, reeds, and hair—from the same eleven graves where maize was recovered. In total, we report 20 AMS radiocarbon dates from these contexts in the S1 Dataset.*

*While we acknowledge the uncertainties of dating burial goods, ceramics and textiles found in the tombs sampled for maize cobs all stylistically date to the pre-Hispanic era. There is no evidence indicating that these tombs were used or visited during modern times; the size and form of the cobs (examples in supplemental figure 1) also suggest all maize are earlier varieties.*

*We acknowledge that it is difficult to determine where maize recovered from archaeological sites was sourced. However, numerous maize cobs were recovered from tombs in the middle valley [7], a context well-suited for maize cultivation. Given the environmental conditions and archaeological context, we are reasonably confident that the maize samples were locally grown. Figure 1 provides the locations of the tombs that were sampled for maize.*

**Finally, the discussion would benefit from exploring why guano fertilization was apparently adopted in the Chincha Valley but not uniformly in neighboring regions, and how adoption patterns may have varied socially or geographically.**

*Thank you for raising these points. To clarify, we are not arguing that guano fertilization was absent or inconsistently adopted in neighboring regions. Rather, in the “Written sources” section, we note that the Lunahuaná people, located in the Cañete Valley north of Chincha, manured their agricultural fields with guano [8]. As discussed in the manuscript, we hypothesize that guano fertilization may have played important roles in the development of multiple coastal Andean societies—including the Moche and Chimú—given both the agricultural challenges of the Andean coast and the accessibility of the Peruvian guano islands. We focus on one area that we feel has a unique geographic and cultural context for guano usage and suggest that our approach provides a promising model for further investigation in other regions.*

*We also address variation in guano acquisition and use among different Andean societies, both in the pre-Hispanic and colonial periods, in the “Written sources” section. We strongly agree that further exploration of social and geographic variation in adoption patterns would be a valuable direction for future research.*

**Editorial Comments:**

**Please ensure that your manuscript meets PLOS ONE's style requirements.**

*We have ensured that the manuscript meets PLOS ONE’s style requirements.*

**If permits were required, please ensure that you have provided details for all permits that were obtained.**

*We wrote the following line in the Acknowledgements: “All necessary permits were obtained for the described study, which complied with all relevant regulations.” A list of the permits we obtained for this study is provided afterwards.*

**Please include a separate caption for each figure in your manuscript.**

*We have included a separate caption for each figure.*

**We note that Figure 1 in your submission contain [map/satellite] images which may be copyrighted.**

*We have replaced Figure 1 with a new map that uses shaded relief and country borders from Natural Earth and Sentinel-2 satellite imagery, which was freely downloaded from https://dataspace.copernicus.eu/.*

*References*

1.	Vaiglova P, Snoeck C, Nitsch E, Bogaard A, Lee‐Thorp J. Impact of contamination and pre‐treatment on stable carbon and nitrogen isotopic composition of charred plant remains. Rapid Communications in Mass Spectrometry. 2014;28: 2497–2510.

2.	Szpak P, Chiou KL. A comparison of nitrogen isotope compositions of charred and desiccated botanical remains from northern Peru. Veg Hist Archaeobot. 2019; 1–12.

3.	Szpak P, Millaire J-F, White CD, Longstaffe FJ. Influence of seabird guano and camelid dung fertilization on the nitrogen isotopic composition of field-grown maize (Zea mays). J Archaeol Sci. 2012;39: 3721–3740.

4.	Szpak P, Longstaffe FJ, Millaire J-F, White CD. Stable isotope biogeochemistry of seabird guano fertilization: results from growth chamber studies with maize (Zea mays). PLoS One. 2012;7: e33741.

5.	Szpak P. Complexities of nitrogen isotope biogeochemistry in plant-soil systems: implications for the study of ancient agricultural and animal management practices. Front Plant Sci. 2014;5: 93091.

6.	Grobman A, Bonavia D, Dillehay TD, Piperno DR, Iriarte J, Holst I. Preceramic maize from paredones and huaca prieta, peru. Proceedings of the National Academy of Sciences. 2012;109: 1755–1759.

7.	Bongers JL. Mortuary practice, imperial conquest, and sociopolitical change in the middle Chincha Valley, Peru (ca. AD 1200–1650). Unpublished PhD dissertation, University of California, Los Angeles. 2019.

8.	De Acosta J. Natural and Moral History of the Indies. Durham: Duke University Press; 2002.

9.	Danielson JJ, Gesch DB, Survey USG. Global multi-resolution terrain elevation data 2010 (GMTED2010). Open-File Report. 2011. doi:10.3133/ofr20111073
