# Supplementary material for: Seabirds shaped the expansion of pre-Inca society in Peru
Source: PLoS One. 2026 Feb 11;21(2):e0341263. doi: 10.1371/journal.pone.0341263 (PMC12893552; doi:10.1371/journal.pone.0341263)
Supplement: Attachment — Submitted filename: Response_to_Reviewers_auresp_2.docx [file pone.0341263.s005.docx]

Responses to Feedback

*We appreciate the insightful feedback from this reviewer, which has improved the paper.*

**Reviewer #3:**

The authors present a study that complements existing knowledge on the use of fertilizers by pre-Hispanic populations. In this regard, the paper’s contribution lies in its focus on a specific area—namely, the Chincha Valley—and its discussion of how fertilization may have supported coastal expansion models.
However some improvements to the manuscripts:

- When the authors refer to Chile, they must specify that it corresponds to the Tarapacá region. Chile is a country over 5,000 km long, with diverse ecological niches, landscapes, and pre-Hispanic cultures. Therefore, to ensure accuracy, it is requested that references to Chile be specified at least in the introduction and background sections as “Tarapacá, Northern Chile” (lines 44, 66, 117, and 272). The archaeological sites of Tarapacá correspond to various locations from the Late Formative, Late Intermediate Period (LIP), and Late Horizon (LH) in Tarapacá.

*Thank you for this point. We have amended the text so that the first mention of the Chilean data now states it is from Tarapacá.*

- The research question clearly addresses the cultural significance and impact of guano use in pre-Hispanic societies.

*We appreciate this assessment!*

- The map should be improved in terms of geographic contextualization. While it shows the spatial relationship between the Chincha Valley, the Tarapacá region, and the sampled sites, it must be enhanced by including information such as a relevant capital city or by adding latitude and longitude coordinates. Additionally, it is requested that the icons representing sampling zones be made clearer and more contrasting.

*We have updated the map to include the capital city of Lima and to improve the contrast of the icons.*

- Given that the sample size is small, clarification is needed regarding the 35 maize cobs mentioned. In Supplementary Figure 1 only fragments of cobs are shown, so it must be clarified whether the 35 cobs refer to complete specimens or fragments. If they are fragments, the authors should explain how the minimum number of 35 specimens was determined, since multiple fragments may belong to the same cob, reducing the actual number of individual samples and the sampling universe. This is essential to clarify due to the statistical analyses being conducted.

I strongly recommend to include some of the morphological characteristics of the archaeological maize samples, especially since not all of them fall within the same range of isotope values

*We have updated the text to clarify (1) the maize cobs are all more than 50% complete (228) and that (2) MNI may be established as the maize fragments were collected from 26 distinct tombs across 14 cemeteries (227-228). This mitigates the issue that the reviewer raises.*

*We appreciate the reviewer’s suggestion to include morphological data for the maize samples. Morphological measurements were not collected for these specimens, although we are considering including them in future studies. We agree that a detailed morphological analysis would be valuable future research. However, the focus for this study is to evaluate isotopic evidence for fertilization practices rather than to characterize the morphology of individual maize samples.*

Results:

- Regarding the material culture findings, the idea is presented that bird iconography on various media may link marine fertility to agricultural productivity. This concept aligns well with Andean logics of duality, complementarity, and reciprocity, but it is not fully developed. It is suggested that the authors expand on this interpretation and revisit the research question by exploring the relationship between coastal resources and agriculture—especially if the aim is to show how seabirds contributed to the development and expansion of human groups in Chincha.

*We agree with the reviewer that, ideally, we would expand the ideological aspects of guano fertilization and its relationship to seabirds in iconography and ethnohistory. However, due to the multidisciplinary approach of this article, and the extensive discussion required for stable isotopic data, we have determined that this is beyond the scope of this article. We have future work planned and would like to include these ideas!*

- It is unclear why the maize cobs were not directly dated. Direct dating of cobs is consistent with the chronology of various Andean archaeological sites, including those in Tarapacá, Northern Chile, referenced in the manuscript (see Vidal et al. 2021, Vidal et al. 2024). The claim that maize cobs may yield misleading radiocarbon results lacks support. Grobman et al 2012´s observation is a specific inconsistency in the Huaca Prieta context and should not be generalized. This point must be clarified, and ideally, the cobs should be directly dated.

*Our maize cobs are dated as best as possible given (1) our available resources and (2) what is presently known regarding radiocarbon dating of uncharred material. We suggest Grobman et al. (2012)’s study indicates caution should be taken when dating uncharred maize cobs, as we have in our sample. While Grobman et al. 2012 found that charred cobs produced radiocarbon results consistent with their stratigraphic context, all* ***nine*** *uncharred cobs, which represent six separate units, produced anomalously young dates, regardless of their stratigraphic positioning. We therefore contend that the issue is not specific to Huaca Prieta, but to the uncharred nature of the material. While we agree that an ideal scenario would involve direct dating of our samples, we do not feel that this is a justified use of resources at this time, as we cannot be sure those dates would provide reliable results––rather, Grobman et al. provide grounds to believe any dates on uncharred cob should be treated with caution. We realize we misstated that some of our cobs were partially charred and have updated the text to record their uncharred nature (556).*

- Although the δ34S results are inconclusive, their inclusion is appreciated and valuable as comparative data for future analyses. This new type of experimental approach is appreciated.

*We appreciate the reviewer recognizing the value of these data to the wider study of maize fertilization.*

- Figures 4, 5, and 6: These figures have low resolution and should be improved to allow proper reading and interpretation.

*We believe the issue has been caused by the file format provided to the reviewer but will ensure high-resolution images are provided for the final document.*

Discussion:

- The relationship between the use of seabird guano as fertilizer and the development of the Chincha Kingdom must be further explored. The manuscript should explore in greater depth how agriculture enhanced by seabird guano fertilization may have contributed to the emergence of complexity in the Chincha Kingdom. For example, was agriculture the foundation for new systems of cooperation or competition? Did it lead to new forms of territorial control? Since this element is central to the research question, it should be critically evaluated, considering relevant archaeological social theory—such as the work of Stanish. The use of the Maritime Foundations of Andean Civilization framework, while appropriate, could be enriched by incorporating more updated perspectives. While the coast is ecologically significant, it is also shaped by human decisions and multiples factors.

*Thank you for raising these thoughtful points. To address how seabird guano fertilization contributed to the development of the Chincha Kingdom, we first must clarify the broader role that manuring can play in driving the expansion of large-scale societies. In the Introduction (lines 57-61), we note that sustainable agricultural practices can promote the expansion of societies by ensuring a stable food supply for growing populations. In the Discussion (lines 478-480), we have revised statements to emphasize that sustainable agricultural practices involving maize production can serve as an economic foundation for large-scale societies by providing reliable food resources that can sustain population growth. We have added the Lombardo et al. (2025) and Finucane (2009) citations to support our argument. Seabird guano fertilization is highlighted as one such sustainable practice, as it directly increases maize crop yields (lines 481-482). We draw attention to evidence of dense Late Intermediate Period and Late Horizon settlement and high population estimates for the late pre-Hispanic Chincha Valley, further demonstrating the potential effects that seabird guano fertilization can have on a large-scale polity such as the Chincha Kingdom (lines 481-484).*

*We provide a detailed discussion about how seabird guano contributed to cooperation and the emergence of complexity in the Chincha Kingdom (lines 493-512). For example, we write about how guano facilitated cooperation between Chincha Kingdom’s specialized farmers, fisherfolk, and merchants. We state, following Curatola’s (1997) model, that fisherfolk sailed to the nearby offshore islands to acquire guano and then subsequently provided it to farmers for enhancing maize production and to merchants for trading along the coast and the highlands, expanding not only Chincha’s agricultural productivity but also its mercantile influence (lines 501-504). This burgeoning network of specialists is a hallmark of the complexity of the Chincha Kingdom. We have added lines 504-507 to note that increased population levels and maize production arising from guano use may have intensified inter-group competition and new forms of territorial control, but these are hypotheses that need to be further explored archaeologically.*

*We agree that the coast is an ecologically important zone that is shaped by human decisions. To this point, we have stated that seabird guano fertilization may have represented an effective regional strategy for producing food despite the challenging, arid Andean coast (lines 454-461 and lines 487-492).*

*We feel we have incorporated Andean work sufficiently and provided a measured interpretation regarding the wider significance to the region while maintaining a manuscript of appropriate length. We feel that additional topics, such as competition, would be better addressed in future follow-up studies looking at wider regional patterns in similar data, and hope such a study will be possible soon. If the reviewer is working on similar research, we encourage them to reach out for a potential collaboration after the publication of this article.*
