# Supplementary material for: Seabirds shaped the expansion of pre-Inca society in Peru
Source: PLoS One. 2026 Feb 11;21(2):e0341263. doi: 10.1371/journal.pone.0341263 (PMC12893552; doi:10.1371/journal.pone.0341263)
Supplement: Attachment — Submitted filename: Response_to_Reviewers_auresp_3.docx [file pone.0341263.s006.docx]

Responses to Feedback

*We appreciate the insightful feedback from this reviewer, which has improved the paper.*

**Reviewer #4:**

- Figure 1 caption, line 119: “Map of Chincha study area, with middle valley cemeteries sampled for maize (marked by yellow squares)” The symbols for maize locations are red/pink circles. I think the yellow squares may have been on a previous version of the map.

*Thank you for pointing this out. We have amended the text to say that the cemeteries are marked by pink circles.*

- Line 110 typo: “however, associated field sprovided inconclusive results for”

*We have revised the text to say that “associated field studies provided inconclusive results for…”*

- Line 115 typo: “marine isotopic values iis lacking”
- Line 128: “We conduct stable carbon, nitrogen, and sulfur analyses” add “isotopic” or “isotope” before “analyses”
- Line 259 spacing typo: “there were no significant changes in δ34S values [47].In this study,”
- Line 396: “terrestrial C4 crops” subscript 4
- Line 490; “cooperation between” should be “among” since there are several groups listed, not two
- Lines 555-556 typo: “The necessity and protocols for pre-treating desiccated and carbonized plants remains debated” add an “is” before “debated”
- Line 638 swap period for comma: “into 8,5 x 5 millimeter”
- Line 640 swap period for comma: “5,5 x 3,5 millimeter tin capsules”

*We are grateful to you for catching these mistakes. They have all been addressed in our manuscript.*

- Line 641 paragraph: be consistent with ‰ and spaces after numbers, as this paragraph has both versions (I would recommend checking the entire manuscript to be consistent)

*Thanks for identifying this inconsistency. We are now consistent with ‰ and spaces after numbers.*

- Line 650 typo?: “n analytical error below 0.1 ‰, 0.2 ‰ and 0.4‰”

*Yes, this is a typo. We have removed ‘n’ from the sentence.*

- Lines 650-654: no superscript for isotopes on these lines
- Line 665: “from middle valley” should this be capitalized?
- Fig 3 caption: “middle Chincha Valley” and “Middle Chincha Valley” both appear (this relates to the capitalization comment earlier)

*We have included superscripts for isotopes on Lines 650-654. In our manuscript, we capitalize ‘Chincha Valley’ when used as part of the phrase ‘middle Chincha Valley;’ otherwise, ‘middle valley’ is not capitalized. This is now consistent in the manuscript.*

- Fig 2 caption: Since Plos One aims for a broad (if mostly academic) audience, should the common names of the birds be included too?

*This is an excellent point. We have now included the common names for the birds in the Figure 2 caption.*

- Fig 6 caption: “C4 crops” subscript 4
- Spacing for ± and numbers is inconsistent throughout the paper

*Thanks again for pointing out these issues. We have corrected the formatting of ‘C_4_’ in Figure 6 and ensured that spacing around the* ± symbol *and numerical values is consistent throughout the paper.*
